# Effect of Meadowsweet Flower Extract-Pullulan Coatings on Rhizopus Rot Development and Postharvest Quality of Cold-Stored Red Peppers

**DOI:** 10.3390/molecules190912925

**Published:** 2014-08-25

**Authors:** Alicja Synowiec, Małgorzata Gniewosz, Karolina Kraśniewska, Anna Chlebowska-Śmigiel, Jarosław L. Przybył, Katarzyna Bączek, Zenon Węglarz

**Affiliations:** 1 Department of Biotechnology, Microbiology and Food Evaluation, Warsaw University of Life Sciences-SGGW, 02-767 Warsaw, Nowoursynowska 159C, Poland; 2 Department of Vegetable and Medicinal Plants, Warsaw University of Life Sciences-SGGW, 02-767 Warsaw, Nowoursynowska 159C, Poland

**Keywords:** pullulan, meadowsweet, antifungal activity, edible coating, pepper

## Abstract

The study involved an examination of the antifungal activity on red peppers of pullulan coating (P) and pullulan coating containing either water-ethanol (P + eEMF) or ethanol extract of meadowsweet flowers (P + eEMF). Pullulan was obtained from a culture of *Aureobasidium* *pullulans* B-1 mutant. Both non-inoculated peppers and those artificially inoculated with *Rhizopus arrhizus* were coated and incubated at 24 °C for 5 days. The intensity of the decay caused by *Rhizopus arrhizus* in the peppers with P and P + eEMF coatings was nearly 3-fold lower, and in the case of P + weEMF 5-fold lower, than that observed in the control peppers. Additionally, the P + weEMF coating decreased, almost two-fold the severity of pepper decay compared to other samples. The influence of coating of pepper postharvest quality was examined after 30 days of storage at 6 °C and 70%–75% RH. All coatings formed a thin and well-attached additional layer of an intensified gloss. During storage, color, total soluble solid content and weight loss of coated peppers were subject to lower changes in comparison with uncoated ones. The results indicate the possibility of the application of pullulan coatings containing MFEs as an alternative to the chemical fungicides used to combat pepper postharvest diseases.

## 1. Introduction

Some biological macromolecules, including polysaccharides, e.g., starches, alginates, celluloses, chitosan and pullulan, are characterized by good coat-forming properties [1,2,3]. The group of bioactive factors which have been incorporated into the coatings include, *inter alia*, organic acids and their salts, enzymes, volatile oils, plant extracts and bioactive compounds isolated from plants [4,5,6,7,8]. An undoubted advantage of such edible coatings is the limited amount of antimicrobial agents transferred to the food, due to their slow release from the coating matrix. Thus, these compounds mainly act on the surface of coated products, *i.e.*, at the site of the highest microbiological food contamination. Antimicrobial coatings may extend considerably the shelf life of raw materials and maintain high levels of food quality during storage in typical cold conditions, as well as during transport and sale [9,10,11].

Pullulan is an exopolysaccharide produced aerobically by the fungus *Aureobasidium pullulans*. It is a non-toxic, non-mutagenic, non-carcinogenic, edible as well as biodegradable polymer with excellent film-forming properties. Pullulan films (usually formed by dried 5%–10% solution of pullulan) are transparent with good oxygen barrier and adhesive properties. Additionally, pullulan films can act as carriers for colors, flavors and other nutritional or antimicrobial additives [12,13].

To date, complex pullulan coatings with incorporated antimicrobials have seldom been used in research studies. These studies have revealed a decline in natural microflora on fresh-cut apples coated with a coating composed of pullulan/glutathione/chitooligosaccharides [14]. Gniewosz and Synowiec [15] focused on pullulan film enriched with thymol against *Bacillus subtilis*, *E. coli*, *Salmonella enteritidis* and *Staphylococcus aureus*. In our previous study, we used a pullulan coating with incorporated extracts of meadowsweet flowers (EMFs) (*Filipendula ulmaria* (L.) Maxim) in order to extend the shelf life of apples [16]. Pullulan coatings containing EMFs not only protected the fruits against bacterial and mold development, but they also contributed to a reduction in weight losses and lower color changes during storage.

Sweet pepper (*Capsicum annuum* L.) is a vegetable cultivated all over the world. Due to their color, taste and nutritional value, peppers are some of the most popular vegetables, and market demand for these vegetables is constantly increasing. Fresh peppers are characterized by a high content of vitamin C and are a good source of carotenoids [17]. However, after harvesting from the plants, the peppers are commonly at risk of undesirable changes, such as microbiological decay (rotting and molding), rapid water loss and ageing, which often cause severe commercial losses [18,19]. One method for the reduction in the effects of these processes may be the coating of the peppers.

Rot of the succulent tissues of vegetables and fruits caused by *Rhizopus* sp. occurs throughout the world. The disease mainly occurs after harvest during the sale, transport, and market storage. *Rhizopus* is omnipresent as a saprophyte and sometimes as a weak parasite on stored organs of plants [20]. Contamination and wounding of fruit during the packing process is the primary source of infection. Symptoms first appear as soft, water-soaked lesions that are not discolored. Lesions develop from wounds, ranging from the stem end or inner walls, and quickly engulf the entire fruit. In storage, these fungi penetrate directly from pips of infected fruit into adjacent healthy fruit [21]. Postharvest diseases of fruits and vegetables caused severe loss of fruit quality [22] and limited pepper fruit export to distant markets [23].

The aim of this study was to determine the antifungal activity on sweet pepper of pullulan coating containing meadowsweet flowers extracts (EMFs), and to evaluate the effect of the coating on the quality of coated peppers during their cold-storage.

## 2. Results and Discussion 

### 2.1. The Effect of Coatings on the Inhibition on Disease Development

**Figure 1:**
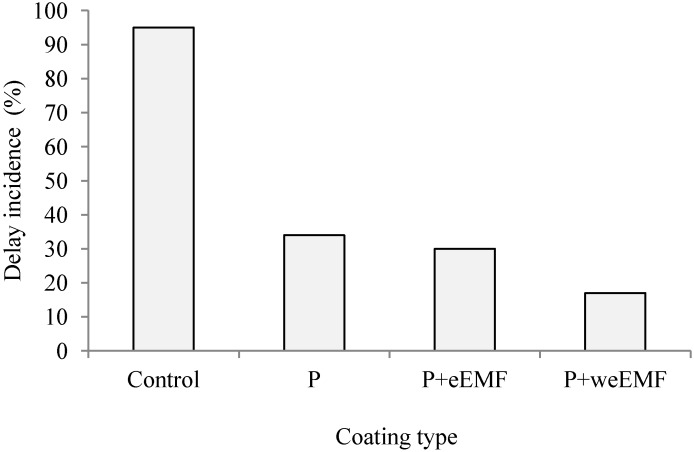
Effect of pullulan coating with or without incorporated meadowsweet flower extract on delay incidence of peppers inoculated with *R. arrhizus* spores after 5 days at simulated marketing conditions (24 °C, RH 55%–60%). The incidence of decay was expressed as a percentage of the peppers exhibiting disease symptoms.

Notes: Uncoated pepper (Control), coated pepper: with pullulan coating (P), with pullulan coating containing ethanol meadowsweet flowers extract (P + eEMF) or with pullulan coating containing water-ethanol meadowsweet flowers extract (P + weEMF).

After 5 days of incubation at 24 °C, it was observed that the coatings applied to peppers limited considerably the incidence of decay in peppers compared to uncoated samples (Figure 1). It may be concluded from our study that the incidence of decay in peppers with pullulan and P + eEMF coatings was about 3-fold lower, and about 5-fold lower in the case of P + weEMF coating compared to the control peppers.

**Figure 2:**
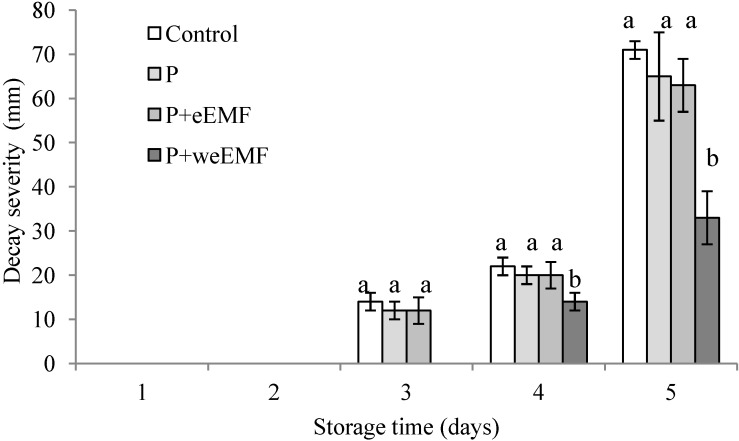
Effect of pullulan coating with or without incorporated meadowsweet flower extract on decay severity of peppers inoculated with *R. arrhizus* spores during 5 days at simulated marketing conditions (24 °C, RH 55%–60%). The severity of decay was expressed as the mean lesion diameter of pepper (mm).

During the 5-day sample incubation period, differentiated disease severity was noted at the site of the cut peel, where peppers had been inoculated with *R. arrhizus* spores (Figure 2). Decay severity on uncoated peppers and on those samples coated with P and P + eEMF coatings was visible from the 3rd day. The severity of decay on coated peppers did not differ significantly statistically from that on uncoated samples. In turn, pullulan coating containing weEMF was the most effective in terms of the inhibition of disease severity of all the examined coatings, and the severity of the decay of the peppers with this coating was significantly lower compared to other samples. The decay severity on those peppers with P + weEMF was visible after 4 days, and after 5 days of incubation the severity of decay was nearly two-fold lower than that observed on other samples.

Uncoated pepper (Control), coated pepper: with pullulan coating (P), with pullulan coating containing ethanol meadowsweet flowers extract (P + eEMF) or with pullulan coating containing water-ethanol meadowsweet flowers extract (P + weEMF). Values are mean ± SD. Columns with different letters (a, b) are significantly different according to Tukey’s test (*p* < 0.05).

### 2.2. The Effect of Coatings on Pepper Quality

#### 2.2.1. General Appearance and Evaluation of the Microstructures of Coated Peppers

**Figure 3:**
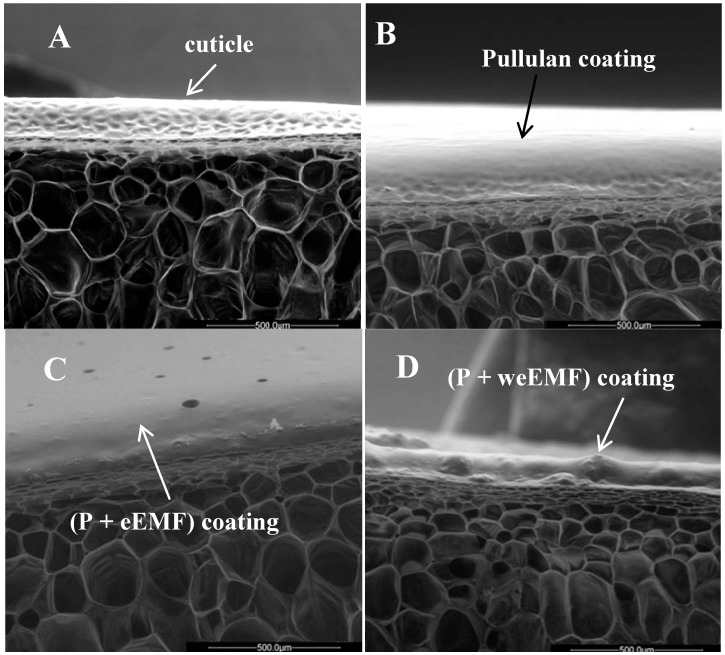
Microscopic image of peel surface and cross-section of uncoated peppers (**A**); and those coated with pullulan coatings enriched with meadowsweet flower extracts (**B**–**D**). The uneven surface cuticle with numerous microhollows in the pepper peel are marked with the arrow (A). The smooth and thin layer of pullulan coating covered all cuticle irregularities (B). Coarse surface of the pullulan coating with meadowsweet flower extracts on the pepper peel are indicated by arrows (C,D).

Pullulan coating or pullulan with EMFs formed a thin, highly adherent additional layer. All coated peppers were characterized by an increase in gloss. The appearance of a fragment of the skins of uncoated and coated peppers is presented in Figure 3. Regular, fine hollows were observed in the cuticule of uncoated peppers (Figure 3A). After an application of pullulan coating on the peppers, these hollows were less visible, which proves they had been filled and their external cuticule sealed (Figure 3B). The surface of the pullulan coating was smooth (Figure 3B), while the surfaces of the coatings with weEMF or eEMF were coarse (Figure 3C,D).

Coating types: (**B**) pullulan coating, (**C**) pullulan coating containing ethanol meadowsweet flower extract (P + eEMF), (**D**) pullulan coating containing water-ethanol meadowsweet flower extract (P + weEMF).

#### 2.2.2. Changes in the Color, Total Soluble Solids (TSS) and Weight Losses of the Peppers

Table 1 presents the values of the color parameters of the peppers. Pullulan coating did not significantly affect the values of parameters *a* * and *b* *. As a result, the color of peppers with pullulan coating did not differ significantly from the color of uncoated peppers. A statistically significant decrease in both parameter *a* * and *b* * was observed as a result of EMF incorporation into pullulan coating. The color of peppers with P + EMFs coatings was 14%–15% less red, and 19%–25% more yellow compared to uncoated peppers.

**Figure 4:**
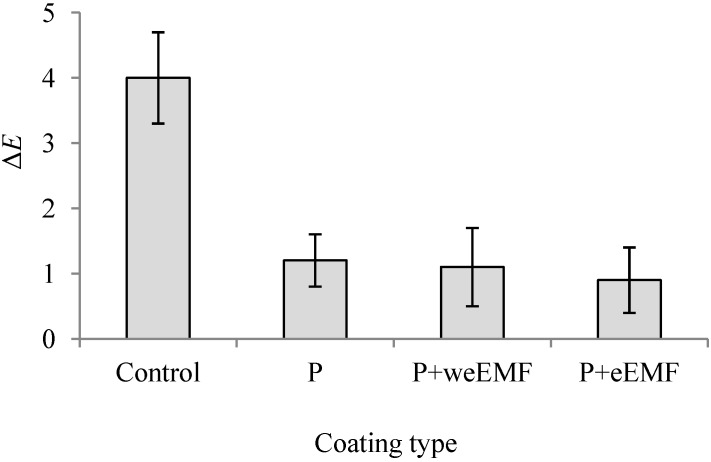
Changes in the color (Δ*E*) of uncoated (Control) and coated peppers after 30 days of cold storage at 6 °C. Red sweet peppers coated with pullulan coating (P), pullulan coating containing water-ethanol meadowsweet flower extract (P + weEMF) or pullulan coating containing ethanol meadowsweet flower extract (P + eEMF). Values are mean ± SD.

Figure 4 presents the changes in the color of peppers after 30 days of storage at 6 °C. It was the most distinct in the case of uncoated peppers (Δ*E* 4.0), which was mainly due to an increase in parameter *a* *. Δ*E* color values were 1.2, 1.1 and 0.9 for the peppers with P, P + weEMF and P + eEMF coatings, respectively.

**Table 1:** Influence of pullulan coating containing meadowsweet flower extract on changes in pepper surface color just after coating. (*L* * = lightness; chromaticity parameters *a* * = redness and *b* * = yellowness).

Color Parameter	Control	P	P + weEMF	P + eEMF
*L* *	33.3 ± 2.4 ^yz^	33.6 ± 1.2 ^yz^	33.1 ± 1.6 ^yz^	30.9 ± 2.2 ^y^
*a* *	22.2 ± 3.7 ^y^	22.0 ± 2.5 ^y^	19.0 ± 2.4 ^z^	18.6 ± 1.8 ^zv^
*b* *	12.4 ± 2.8 ^y^	11.9 ± 1.0 ^yz^	10.0 ± 1.3 ^v^	9.6 ± 1.3 ^v^

Uncoated peppers (Control), coated peppers: pullulan coating (P), pullulan with water-ethanol meadowsweet flower extract (P + weEMF), pullulan with ethanol meadowsweet flower extract (P + eEMF). Values are mean ± SD. Different superscript letters (y, z, v) within the same row indicate significant differences of means (*p* < 0.05). The mean values were compared as per Tukey one-way analyses of variance.

**Figure 5:**
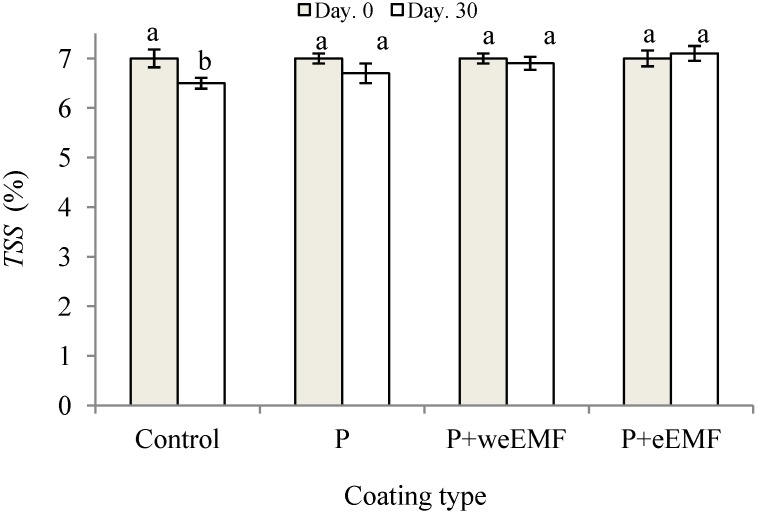
Changes total soluble solids contents (TSS) in uncoated (Control) and coated peppers after 30 days of cold storage at 6 °C. Red sweet peppers coated with pullulan coating (P), pullulan coating containing water-ethanol meadowsweet flower extract (P + weEMF) or pullulan coating containing ethanol meadowsweet flower extract (P + eEMF). Values are mean ± SD. Columns with different letters (a, b) are significantly different according to Tukey’s test (*p* < 0.05).

As shown in Figure 5, the content of total soluble solids (TSS) decreased slowly in the peppers during 30 days of storage at 6 °C. The content of TSS in uncoated peppers decreased gradually from an initial value of 7.0% to 6.5% after 30 days. In turn, TSS content in coated peppers at the end of storage was 6.7%, 6.9% and 6.8% for the peppers with P, P + weEMF and P + eEMF coatings, respectively. No significant differences in TSS content in the coated samples were noted during the storage.

Weight losses were observed during storage of the peppers (Table 2). The highest losses of sample weight were noticeable during the first two days of the storage, and these were 3.2% for uncoated peppers, and 2.6%, 1.8% and 1.5% for those peppers with pullulan, P + weEMF and P + eEMF coatings, respectively. Total weight loss in the case of uncoated peppers was 13.5%, while for coated ones it was 12.6%–12.8%.

The changes of peppers weight loss during storage was described by regression equations. The parameters of equations and coefficients of determination are given in Table 3. Uncoated peppers exceed the maximum permissible weight loss (5%) after 6.3 days of storage and those coated with pullulan coating with or without EMFs exceed the permissible weight loss after 7.8 days. According to this assumption, peppers coated with pullulan coating were able to store for an additional 1.5 days.

**Table 2:** Changes in weight losses (%) in the peppers stored at 6 °C during 30 days.

Storage Day	Control	P	P + weEMF	P + eEMF
2	3.2 ± 0.2 ^a^	2.62 ± 0.16 ^b^	1.81 ± 0.14 ^c^	1.51 ± 0.20 ^c^
4	3.9 ± 0.1 ^a^	3.50 ± 0.18 ^b^	3.31 ± 0.22 ^b^	3.3 ± 0.20 ^b^
6	5.13 ± 0.12 ^a^	4.4 ± 0.12 ^b^	4.37 ± 0.20 ^b^	4.45 ± 0.23 ^b^
8	5.42 ± 0.20 ^a^	4.60 ± 0.17 ^c^	4.90 ± 0.17 ^b^	4.82 ± 0.21 ^b^
10	6.50 ± 0.10 ^a^	5.81 ± 0.31 ^b^	5.93 ± 0.30 ^b^	6.01 ± 0.12 ^b^
12	6.91 ± 0.09^a^	6.17 ± 0.20 ^b^	6.61 ± 0.21 ^b^	6.51 ± 0.17 ^b^
14	7.94 ± 0.10 ^a^	7.22 ± 0.12 ^b^	7.37 ± 0.17 ^b^	7.50 ± 0.21 ^b^
16	8.50 ± 0.30 ^a^	7.63 ± 0.10 ^c^	8.05 ± 0.15 ^b^	8.03 ± 0.20 ^b^
18	8.90 ± 0.10 ^a^	8.39 ± 0.13 ^b^	8.50 ± 0.21 ^b^	8.51 ± 0.24 ^b^
20	9.91 ± 0.11 ^a^	9.31 ± 0.15 ^b^	9.41 ± 0.20 ^b^	9.60 ± 0.22 ^b^
22	10.78 ± 0.15 ^a^	10.22 ± 0.20 ^b^	10.22 ± 0.25 ^b^	10.42 ± 0.19 ^b^
24	11.63 ± 0.12 ^a^	11.34 ± 0.20 ^b^	10.90 ± 0.12 ^c^	10.62 ± 0.11 ^d^
26	12.79 ± 0.22 ^a^	12.07 ± 0.15 ^b^	11.62 ± 0.15 ^c^	11.55 ± 0.20 ^c^
28	13.12 ± 0.20 ^a^	12.20 ± 0.12 ^b^	12.25 ± 0.10 ^b^	12.63 ± 0.11 ^c^
30	13.55 ± 0.13 ^a^	12.61 ± 0.16 ^b^	12.80 ± 0.13 ^b^	12.70 ± 0.12 ^b^

Notes: Uncoated peppers (Control), coated peppers: pullulan coating (C), pullulan coating containing water-ethanol meadowsweet flower extract (P + weEMF), pullulan coating containing ethanol meadowsweet flower extract (P + eEMF). Values are mean ± SD. Different superscript letters (a, b, c, d) within the same row indicate significant differences of means (*p* < 0.05). The mean values were compared as per Tukey one-way analyses of variance.

**Table 3:** The parameters of the regression equation describing the change of peppers weight loss during storage and the calculated storage time in which pepper achieve the maximum permissible weight losses of 5%.

Coating Type	Regress Equation *y = ax + b*			Storage Time (days)
	*R^2^*	*a*	*b*	
Control	0.973	0.402	1.952	6.3
P	0.980	0.392	1.477	7.7
P + weEMF	0.982	0.396	1.413	7.8
P + eEMF	0.978	0.400	1.363	7.8

*y*—weight loss [%]; *x*—storage time [days].

### 2.3. Discussion

Due to the increasingly widely discussed issues concerning the safety of the chemical preservatives used in the extension of food shelf life, the interest in antimicrobial coatings containing natural preservatives has been growing [24]. The influence on mold growth inhibition of pullulan coating with incorporated EMFs was differentiated. Mold development on coated peppers was probably a result of the absence of coating continuity at the site of inoculation. Neither a too thin nor a non-uniform coating exhibited an inhibiting activity towards mold, the development of which caused pepper decay almost to the same degree as in the case of uncoated peppers. Stronger antifungal activity was noted in the case of pullulan coating with weEMF compared to eEMF, which probably resulted from the different active compound content observed in the examined extracts. It was concluded from our previous research that the content of bioactive compounds, including phenolic acids and flavonoids, was statistically significantly higher in weEMF than in eEMF [16]. It may be reasoned from the previous studies of other authors, that the effectiveness of coating activity depends on their composition, and multi-component coatings exhibit stronger antimicrobial activity compared to mono-component ones. Maqbool *et al.* [25] observed a stronger inhibitory effect of anthracnose on banana coated with gum Arabic with incorporated chitosan compared to banana coated with coatings with these components incorporated separately. It has been demonstrated in other studies that the incorporation of volatile oils into a chitosan coating may considerably improve its antibacterial properties [25,26,27]. Palou *et al.* [28] claim that the effectiveness on fresh fruits and vegetables of coatings with various incorporated antifungal components is usually limited to fungistatic rather than to fungicidal activity. Shao *et al.* [29] observed a strong limitation in the growth of *Penicillium expansum* and *Botrytis cinerea* fungi on apples coated with chitosan; however, they noted that the coating did not entirely inhibit their development.

It was observed that the coating was only formed on the surface of the examined samples and did not penetrate inside, due to protective action of the hydrophobic components the cuticule is impregnated with: cutin and intracuticular waxes [30]. An introduction of EMFs to the pullulan coating increased its thickness [16], causing a formation of an even, uniform layer (Figure 3C,D). Due to the high degree of adhesion to the cuticule, the coating was an additional physical barrier. Our observations are consistent with the reports of Conforti and Totty [9], who claim that the coatings primarily increase fruit peel resistance. Fruit coating causes a blocking of part of the pores occurring in the skin, which results in an inhibition of the diffusion of gases and slowed fruit respiration.

Pullulan coating and pullulan with weEMF did not change the color brightness of the coated peppers, while coating containing eEMF caused color darkening compared to uncoated peppers. A decrease in pepper color lightness (*L* *) may be perceived as their browning [31]. After storage at 6 °C for 30 days, the color of peppers was subject to the change (Figure 4). It was the most distinct in the case of uncoated peppers, which was mainly due to an increase in parameter *a* *. This change proved both an increase in carotenoid content in the peppers and their maturation [32]. In turn, the color of coated peppers was subject to slight changes. Such low color changes during the storage of coated peppers may on the one hand prove the profitable effect of the coatings in the inhibition of maturation processes, but on the other hand the masking of these processes by the color of EMFs introduced to the pullulan coating. In addition, the changes in the content of total soluble solids (TSS) and weight loss were determined in the peppers during 30 days of storage at 6 °C (Figure 4 and Table 2).

The main changes in vegetable composition during storage period are usually associated with ripening, respiration and moisture evaporation [33]. In this work coating containing weEMF reduced weight loss and *TSS* content of coated peppers during storage compared to uncoated samples, which indicates to slow down of respiration and metabolic changes retarding the ripening process [34,35]. Similar effects were observed for guava fruit coated with chitosan coating [36], kiwi fruits coated with a mixture of soy proteins, pullulan and SA [37], or strawberries coated with a mixture of chitosan (1%) and oleic acid (2%) [38].

Weight loss from the peppers is the main factor reducing their quality and long-term storage. The rate of postharvest weight losses in pepper fruits during storage determines the loss of freshness, durability time is subject to shortening and economic value loss is observed [18]. Compared to other fruits of a similar shape and size, the pepper is characterized by a large surface (SA) to weight (FW) ratio, since the fruits are empty inside [30], which means that peppers are particularly susceptible to weight losses [39]. Successive weight losses were observed during storage of the peppers (Table 2). Pullulan coating (with and without the addition of extracts) which protected pepper peels, did not limit weight losses during storage, due to the natural processes of water loss occurring in various parts of the fruit. Ben-Yehoshua and Rodov [33] revealed that water loss from the fruits occurs not only through the stomata, lenticels, cuticle, and epicuticular wax platelets, but also through the calyx, pedicel or floral ends. Díaz-Pérez *et al.* [39] examined the permeability of water vapor through various parts of the pepper, and they noted that it was about 14% lower through the peel compared to the fruit calyx. The authors noted that about 26% of water losses in mature peppers occurred through the calyx. The results of these reports may explain the reason for low level of protection offered to peppers by the coatings applied, which only protected the cuticule against water loss, but not the calyx of the peppers. The stemscar or calyx may also be a site of intense oxygen, ethylene and carbon dioxide diffusion in the fruits. Díaz-Pérez *et al.* [39] noted that as much as 80%–90% of the diffusion of these gases in the peppers occurs through the pedicels and stemscars. Based on the results of the study, the authors estimated that the maximum allowable losses in pepper weight during storage should not exceed 4.5%–5%, which may be obtained during 8–10 days of pepper storage at 7–10 °C and RH 85%–90%. In view of our estimations, the application of complex pullulan coatings prolonged pepper postharvest life from 6 to 8 days of storage at 6 °C and RH 70%–75%.

## 3. Experimental Section

### 3.1. Materials

Pullulan was obtained from a culture of white mutant *A. pullulans* B-1 following the protocol described in an earlier work [40,41]. The pullulan content in the raw preparation was 72% and this was determined according to the procedure described by Güksungur *et al.* [42]. Meadowsweet flowers (*Filipendula ulmaria* (L.) Maxim) were harvested from a natural site located on a humid post-peat bog meadow in north-eastern part of Poland (N 52 44.122' E 022 48.803'). Meadowsweet flower extracts (EMFs) were prepared using the extraction method, which was described in an earlier work [16]. Two liquid extracts were prepared: ethanol (eEMF) of a density of 0.28 g d.w./mL, and a water-ethanol mixture (weEMF) of a density of 0.50 g d.w./mL.

Red sweet peppers (*Capsicum annuum* L. cv. Salomon) for which the producer declared not using any postharvest chemical treatments were purchased from a local shop in Warsaw (Poland). The peppers were sorted after their delivery to the laboratory. Those peppers with any external injuries or diseases, and similar in terms of maturity and size, were selected for the study.

### 3.2. Coat-Forming Solutions

The pullulan solution contained [g d.w./100 mL]: raw pullulan preparation 7.5, glycerol 3.0 and distilled water up to 100 mL. Then, we prepared pullulan solutions containing EMFs of the following composition [g/100 mL]: raw pullulan preparation 7.5, glycerol 3.0, weEMF or eEMF 12.0 and distilled water up to 100 mL. Glycerol (POCH S.A., Gliwice, Poland) was used as a plasticizer in order to improve coating flexibility. Pullulan and glycerol were dissolved in hot distilled water (80 °C). EMFs were added to the pullulan solution after cooling to the room temperature.

### 3.3. Fungal Inoculum

*Rhizopus arrhizus* ATCC 11145 is a saprophyte commonly observed on numerous vegetable species. *R. arrhizus* is a fast-growing fungus with low requirements and causing fruit decay. The strain was obtained from the pure culture collection of the Department of Biotechnology and Food Microbiology (WULS-SGGW, Warsaw, Poland). The strain was cultured for 14 days on PDA in Petri dishes at 24 °C. A suspension of mold spores was prepared in physiological saline (NaCl 0.9%, w/v) with an addition of Tween 80 (Sigma-Aldrich Co., Poznań, Poland) (0.05%, w/v). The number of spores was calculated using a hemocytometer. The suspension was diluted to the desired inoculum density of 10^5^ spores/mL using sterile water.

### 3.4. Fruit Inoculation and Coating Application

The peppers were disinfected by immersion them in sodium hypochlorite (POCH S.A.) (0.05% w/v) for 2 min, and then rinsed in sterile water for 2 min, and left at room temperature (24 °C) until completely dry. Afterwards, a cross-shape of a length of 20 mm and at a depth of about 1 mm was cut in the peel of each pepper with a disinfected knife. The samples were immersed in *R. arrhizus* spores suspension (10^5^ spores/mL) for 2 min and left at room temperature for 15 min for drying. Then, a suitable coat-forming solution was sprayed on the whole pepper surface using a PZ-270XS aerograph (with a 0.5 mm nozzle manufactured by PointZero Airbrush Company, Seattle, WA, USA); two layers were applied. Coated peppers were left in a laminar chamber for 2 h at 24 °C and RH 55%–60%. Inoculated, but non-coated peppers were used as a control. Four groups of inoculated peppers were prepared: coated with a pullulan coating (P), pullulan with water-ethanol EMF (P + weEMF), pullulan with ethanol EMF (P + eEMF) and uncoated ones (Control). Each group consisted of thirty peppers (10 peppers in three reps). The fruits were placed on plastic trays and stored for 5 days (24 °C, RH 55%–60%) in simulated market conditions. The effect of pullulan coating, both with and without incorporated EMFs, on the incidence of decay and also the severity of the decay in the peppers was evaluated daily for 5 days. The incidence of decay was assessed visually and expressed as a percentage of the peppers exhibiting disease symptoms. The severity of decay was expressed as the mean lesion diameter. The diameter of pepper decay was measured from the site of the pepper peel cut and expressed in mm.

### 3.5. Evaluation of Quality Parameters

#### 3.5.1. Pepper Coating and Storage

The peppers were disinfected by immersing in sodium hypochlorite (0.05%, w/v) for 2 min, and then rinsed in sterile water for 2 min and left at room temperature (24 °C) until completely dry. Then, the peppers were coated in the way described above (Section 3.4). The peppers were divided into 4 groups, thirty fruits in each; *i.e.*, coated with pullulan coating (P), pullulan with water-ethanol EMF (P + weEMF), pullulan with ethanol EMF (P + eEMF) and uncoated ones (Control). The samples were stored for 30 days in open boxes at 6 °C, RH 70%–75% in a refrigerator of the Department of Biotechnology and Food Microbiology (WULS-SGGW, Warsaw, Poland).

#### 3.5.2. Microstructure Observations

The microstructures of the surfaces and cross-sections of peppers peels in each group were examined using a scanning electron microscope (SEM) (type FEI, Quanta 200, Jeol Ltd., Tokyo, Japan). Observations and micrographs were performed in low vacuum mode (LV mode) using an LFD detector, at an accelerating voltage of 30 kV.

#### 3.5.3. Color Determination

Color CIELAB parameters (*L* *, *a* *, *b* *) were determined using a spectrophotometer (Model CM-3600D, Minolta Camera Co., Osaka, Japan), using as measurement conditions the illuminant D65/observer 10°, with a diaphragm MAV 4 mm. The values of *L* * (lightness), *a* * (redness), and *b* * (yellowness) were registered in order to evaluate the changes in pepper surface colors on the first day after coating application (*L*_0_, *a*_0_, *b*_0_) and on the last day of material storage (*L*_1_, *a*_1_, *b*_1_). Each group included ten peppers allocated solely for this purpose. Three analyses of color along the equatorial axis were conducted on each pepper.

Additionally, the total color difference (TCD) value (Δ*E*), which considers the changes in all coordinates, was calculated according to the following equation [43]:

Δ*E* = [(*L*_1_ − *L*_0_)^2^+(*a*_1_ − *a*_0_)^2^ + (*b*_1_ − *b*_0_)^2^]^0.5^ (1)

#### 3.5.4. Pepper Weight Loss and Total Soluble Solids Evaluations

The peppers were weighed every two days over the whole period of sample storage. The results were expressed as the percentage loss of initial weight. The total soluble solid contents (TSS) of the non-diluted juice from five peppers was determined at 20 °C by using a refractometer (Model DR-103L, Index Instruments Limited, Ramsey, U.K.). Data was expressed in degrees Brix. Peppers were homogenised using a kitchen blender and then a drop of juice was placed on the prism glass of the refractometer to obtain the (Brix) reading.

#### 3.5.5. Determine the Prolongation of Postharvest Life of Pepper

To determine the prolongation of postharvest life of the coated peppers, the maximum permissible weight losses of 5% was used according to Díaz-Pérez *et al.* [39]. After the fruit lost about 5% of their initial weight, peppers lose their firmness, which becomes noticeable by consumers and therefore are not allowed in retail sales. The regress equation was calculated, afterwards the time was calculated after which the weight loss of peppers reach 5%.

### 3.6. Statistical Analysis of the Results

Statistical tests were performed using the Statistica computer program (version 10PL, StatSoft Inc., Krakow, Poland). One-way analysis of variance was performed. The significance of differences between mean values was assessed using the Tukey-test at a significance level of *p* < 0.05.

## 4. Conclusions

In summary, the application of complex edible pullulan coating with meadowsweet flowers extracts is a simple way to decrease losses caused by fungal development on peppers. The results of the present study showed that pullulan film containing water-ethanol extract from meadowsweet flowers exhibited the strongest antifungal activity. The application of this coating onto peppers surface reduced the peppers decay and decreased the disease incidence caused by Rhizopus rot. These coatings are easily formed on the peppers, providing a glossy, slightly darker color. An additional advantage of peppers coating was the reduction in the content of total soluble solids during their storage, as well as the prolongation of the postharvest life of the peppers. Such coatings may be used as an alternative to the commercial chemical fungicides used for the control of fungicidal contamination on vegetables.
